# Preparation of Fe/PANI/GF Composite and Study on Its Interfacial Evaporation Performance

**DOI:** 10.3390/nano16010024

**Published:** 2025-12-24

**Authors:** Jipu Guo, Xiaolong Wei, Meiyan Wang, Xu Li, Bin Yan, Xiaotong Pan, Zhe Zhang, Yao Wu, Bofang Shi, Honghui Yang

**Affiliations:** 1 State Grid (Xi’an) Environmental Protection Technology Center Co., Ltd., Xi’an 710100, China; jipu_guo@sina.com (J.G.);; 2 State Grid Shaanxi Electric Power Company Xi’an Power Supply Company, Xi’an 712000, China; 3 State Grid Shaanxi Electric Power Co., Ltd. Electric Power Science Research Institute, Xi’an 710100, China; 4 School of Chemistry, Xi’an Jiaotong University, Xi’an 710049, Chinabofangshi@xjtu.edu.cn (B.S.)

**Keywords:** interfacial evaporation, polyaniline (PANI), graphite felt (GF), photothermal material, seawater desalination

## Abstract

Addressing the global shortage of freshwater resources necessitates the development of efficient and economical photothermal evaporation materials. Herein, an Fe/polyaniline/graphite felt (Fe/PANI/GF) composite was fabricated by combining electrochemical deposition and impregnation. The structural characteristics, photothermal conversion efficiency, and interfacial evaporation performance of the composite were systematically investigated. Results demonstrate that Fe/PANI/GF exhibits a remarkably high solar absorption rate of 95% across the 300–2000 nm wavelength range. Under 1 kW m^−2^ illumination, the surface temperature of Fe/PANI/GF rapidly increased from ambient temperature to 60.3 °C within 5 min. The composite achieved an evaporation rate of 2.05 kg m^−2^ h^−1^, corresponding to an interfacial evaporation efficiency of 70.3%. This exceptional performance is attributed to the synergistic effect between the broad-spectrum light absorption of PANI and the enhanced light absorption induced by Fe coordination, which collectively promote the photothermal conversion process. This study provides valuable insights for the development of high-performance solar interfacial evaporation materials.

## 1. Introduction

Escalating global energy demand, combined with worsening environmental pollution, underscores the critical need for clean and sustainable energy solutions to support the low-carbon transition of human society [1,2]. As an abundant, renewable, and clean energy resource, solar energy has witnessed the maturation of its conversion and utilization technologies into a relatively well-established system via continuous innovation [3,4]. Solar-driven interfacial evaporation (SDIE) technology, leveraging its unique energy utilization mechanism, exhibits significant potential in both academic research and industrial applications. By precisely confining the evaporation process to the water/air interface, SDIE effectively minimizes bulk heat conduction losses inherent in traditional evaporation methods, thereby achieving a substantial improvement in evaporation efficiency. This technology holds considerable promise for applications such as seawater desalination, industrial wastewater treatment, and water resource recycling [5,6,7,8].

Within SDIE systems, photothermal materials serve as the core component, whose performance directly determines the evaporation efficiency and application efficacy of the system. These materials are typically constructed as an absorbing layer at the water/air interface to drive water evaporation via the photothermal conversion effect [6,9]. Recent research efforts have focused on enhancing the solar absorption rate and photothermal conversion efficiency of photothermal materials, leading to the development of various novel photothermal material systems. Among these, conjugated polymer materials, represented by polyaniline (PANI), have emerged as a research hotspot due to their excellent photothermal conversion performance and environmental stability [10,11,12]. From a microstructural perspective, the linear conjugated π-electron system in PANI molecules endows them with high electron mobility. The absorption spectrum red-shift induced by conjugated π-bonds significantly enhances the material’s light absorption capability in the near-infrared (NIR) region. Concurrently, protonic acid doping effectively reduces the bandgap of PANI, thus facilitating the transition of excited electrons between the valence and conduction bands, thereby further improving light absorption efficiency [13,14,15]. Furthermore, coordination of transition metal ions with PANI can alter the electronic structure of PANI, enhancing its light absorption and providing a stronger foundation for photothermal conversion [16,17].

Building on this foundation, this study proposes the construction of a novel Fe/PANI/GF composite material through Fe-N coordination chemistry. This material integrates high-efficiency photothermal conversion performance with excellent potential for application in solar interfacial evaporation. This paper systematically investigates the preparation process, microstructural characteristics, and performance of Fe/PANI/GF in solar interfacial evaporation applications. The aim is to provide an efficient and economical photothermal material solution for the advancement of solar desalination technology, thus facilitating the industrialization of SDIE.

## 2. Materials and Methods

### 2.1. Materials and Instruments

#### 2.1.1. Main Materials

Graphite felt (GF, 2 mm) was purchased from (Beihai Carbon Co., Ltd., Beihai, China) Aniline, sulfuric acid (H_2_SO_4_), sodium hydroxide (NaOH), absolute ethanol, and ferrous sulfate heptahydrate (FeSO_4_·7H_2_O) were obtained from (Sinopharm Chemical Reagent Co., Ltd., Shanghai, China). All reagents were used as received without further purification.

#### 2.1.2. Main Instruments

Muffle furnace (CMF-1100, Hefei Kejing Materials Technology Co., Ltd., Hefei, China); Electrochemical workstation (CHI660e, Shanghai Chenhua Instrument Co., Ltd., Shanghai, China); DC power supply (IT6720, ITECH Electronic Co., Ltd., Nanjing, China); Xenon lamp light source (PLS-SXE300+/UV, Beijing PerfectLight Technology Co., Ltd., Beijing, China); Electronic balance (ME204E, Mettler Toledo, Greifensee, Zurich, Switzerland).

### 2.2. Preparation of Fe/PANI/GF

#### 2.2.1. Pretreatment of GF

Commercially available GF was cut into pieces measuring 2.5 cm × 5 cm. The GF pieces were sequentially ultrasonically cleaned with absolute ethanol and ultrapure water for 20 min each to remove surface impurities, followed by drying at 60 °C for 2 h to obtain clean GF. The clean GF was then placed in a muffle furnace. The temperature was raised from 30 °C to 450 °C at a rate of 5 °C min^−1^ and maintained at 450 °C for 2 h to obtain activated GF [18].

#### 2.2.2. Preparation of PANI/GF Composite

PANI/GF composites were prepared using a constant current method in a two-electrode system [19]. The activated GF served as both the cathode and anode. Electrodeposition was performed in an electrolyte solution containing 0.1 M aniline and 0.5 M H_2_SO_4_ for 10 min at current densities of 0.25, 0.5, 1, 2, 5, and 10 mA cm^−2^. After washing and drying, emeraldine salt PANI (PANI_ES_/GF) was obtained. Emeraldine base PANI (PANI_EB_/GF) was prepared by stirring PANI_ES_/GF in 0.2 M NaOH solution for 30 min.

#### 2.2.3. Preparation of Fe/PANI/GF Composite

The Fe/PANI/GF composite was prepared via a simple impregnation method [20]. The cleaned PANI_ES_/GF was immersed in a 0.12 M FeSO_4_ solution and stirred for 2 h. The sample was then thoroughly washed with ultrapure water to completely remove free Fe ions; 1,10-phenanthroline was used to detect residual Fe ions in the wash solution. The prepared Fe/PANI/GF sample was dried and stored at room temperature for subsequent use.

### 2.3. Material Characterization

The morphology of the photothermal graphite felts was observed using scanning electron microscopy (SEM, EVO10, Beijing Saier Network Co., Ltd., Beijing, China). Elemental composition analysis was performed using an attached energy dispersive spectrometer (EDS). Fourier transform infrared spectroscopy (FT-IR, VERTEX70, Shaanxi Yingmei Electronic Technology Co., Ltd., Xi’an, China) was conducted within the wavenumber range of 400-4000 cm^−1^. Contact angle measurements were performed using an optical contact angle meter (DSA100S, KRÜSS, Hamburg, \Germany). Reflectance spectra in the wavelength range of 300-2000 nm were obtained using a UV-Vis-NIR spectrophotometer equipped with a 150 mm integrating sphere (PE Lambda950, Beijing Saier Network Co., Ltd.). Surface temperature changes during evaporation were recorded using an infrared thermal imager (THT45W, HT, Faenza, Italy).

### 2.4. Evaporation Performance Test

A xenon lamp light source equipped with an AM 1.5G filter was used as the simulated solar light source, and the light intensity was calibrated using an optical power meter. Indoor evaporation experiments were conducted at a constant temperature of 25 ± 1 °C and relative humidity of 40 ± 5%. The photothermal graphite felt and an EPS foam support were fixed at the mouth of a beaker, with two edges of the graphite felt penetrating the foam to immerse in water. The xenon lamp illumination was focused on the center of the graphite felt. Continuous irradiation was applied for 1 h, and mass data were recorded every 10 min using an analytical balance. Three parallel experiments were conducted for each sample.

The water evaporation rate (*v*) was calculated as follows:

(1) $$v=\frac{\Delta m}{\mathrm{St}}$$

where *v* is the water evaporation rate (kg m^−2^ h^−1^), *Δm* is the mass change (kg), *S* is the illuminated area of the photothermal graphite felt (m^2^), and *t* is the illumination time (h).

The evaporation efficiency (*η*) was calculated as follows:

(2) $$\eta =\frac{\dot{m}\left(L_{v}+Q\right)}{C_{\mathrm{opt}}P_{0}}$$

(3) $$L_{v}+Q=\frac{1}{1000}(C_{p}\Delta T+\Delta H_{\mathrm{vap}})$$

where *η* is the evaporation efficiency (%), *ṁ* is the steady-state water evaporation rate (kg m^−2^ h^−1^), *L_v_* is the sensible heat of water evaporation (kJ kg^−1^), *Q* represents the latent heat of water evaporation (kJ kg^−1^), *C_opt_* is the optical concentration factor at the graphite felt surface (dimensionless, representing multiples of the standard solar irradiance), *P*_0_ is the solar irradiance (1 kW m^−2^), *C_p_* is the specific heat capacity of water (4.18 kJ kg^−1^ K^−1^), Δ*T* is the surface temperature difference in the graphite felt during evaporation (K), and Δ*H_vap_* is the enthalpy of vaporization of water (kJ mol^−1^) [21].

## 3. Results

### 3.1. The Optimization of Electrodeposition Current Density for PANI/GF and Its Effect on Evaporation Performance

**Figure 1:**
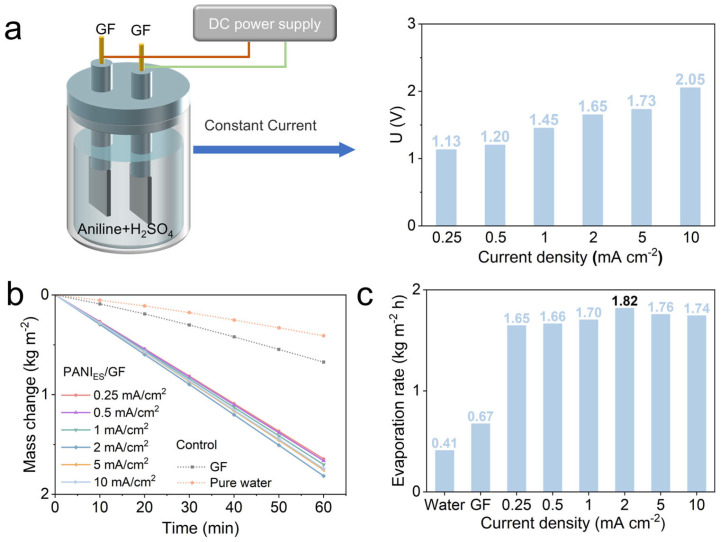
Optimization of PANIES/GF preparation conditions. (**a**) Voltage variation of PANI_ES_/GF at different current densities; (**b**,**c**) Evaporation rate of PANI_ES_/GF at different current densities.

The constant-current electrodeposition process is critical for regulating the structure and performance of PANI/GF, as current density directly affects PANI nucleation, coating uniformity, and subsequent photothermal evaporation efficacy. To optimize this parameter, the electrochemical behavior during PANI deposition and the evaporation performance of the resulting PANIES/GF composites were systematically investigated (Figure 1). As shown in Figure 1a, the steady-state cell voltage exhibited a monotonic increase with rising current density, ranging from 1.13 V at 0.25 mA cm^−2^ to 2.05 V at 10 mA cm^−2^. This trend is attributed to increased charge transfer resistance at higher current densities: accelerated PANI polymerization under high current may cause uneven nanoparticle growth on GF fibers, whereas low current densities result in insufficient PANI loading—both of which can compromise material performance.

The impact of current density on evaporation performance was further verified via solar-driven evaporation tests (Figure 1b). Pristine GF showed a low water mass loss rate (corresponding to 0.67 kg m^−2^ h^−1^), while pure water and the blank control exhibited negligible evaporation (0.41 kg m^−2^ h^−1^) due to limited solar absorption capacity. For PANI_ES_/GF, evaporation performance first improved with increasing current density, peaking at 2 mA cm^−2^ (mass loss rate corresponding to 1.82 m^−2^ kg^−1^ h^−1^). At higher densities (5-10 mA cm^−2^), performance slightly decreased.

Based on these results, 2 mA cm^−2^ was selected as the optimal electrodeposition current density for PANI_ES_/GF preparation, ensuring balanced PANI coating uniformity and photothermal activity for subsequent Fe coordination.

### 3.2. Morphology and Characterization of GF, PANI/GF, and Fe/PANI/GF Composites

**Figure 2:**
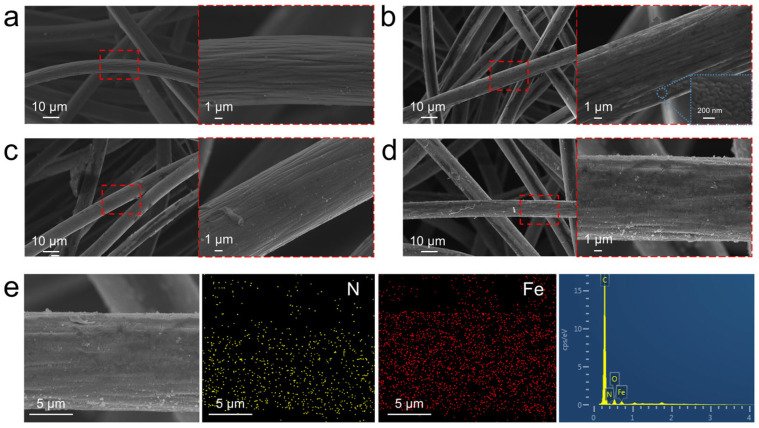
Morphologies of the GF composites. SEM image of (**a**) GF; (**b**) PANI_ES_/GF; (**c**) PANI_EB_/GF; (**d**) Fe/PANI/GF; (**e**) EDS elemental mapping of Fe/PANI/GF.

The micro-morphologies of the different photothermal graphite felts were characterized by SEM, as shown in Figure 2. The pristine GF exhibited a typical three-dimensional interconnected fibrous network structure with smooth fiber surfaces and an average diameter of approximately 10 μm (Figure 2a). After loading PANI onto the GF surface via electrodeposition, the PANI forms uniform nanoparticles with an average diameter of 80±10 nm on GF fibers. PANI was tightly anchored onto the GF fiber surfaces, significantly increasing surface roughness (Figure 2b). This structural feature enhances surface hydrophilicity and light trapping capability through the introduction of polar groups (e.g., -NH-) [22,23]. For the Fe/PANI/GF composite obtained through Fe coordination, SEM images revealed a further increase in the surface roughness of GF. No significant agglomeration of Fe was observed (Figure 2d), indicating the formation of a stable interfacial bond between Fe and PANI.

To confirm the elemental composition and distribution of Fe/PANI/GF, energy-dispersive spectroscopy (EDS) analysis was conducted (Figure 2e). The results showed that Fe (red) and N (blue) elements were uniformly distributed on the fiber surface, confirming the successful loading of Fe onto the PANI/GF system through coordination, forming a stable composite structure. EDS elemental analysis shows that Fe/PANI/GF consists of 82.4 wt% C (from GF), 1.52 wt% N (from PANI), 10.96 wt% Fe (from Fe coordination), and 5.13 wt% O (from surface oxidation), consistent with the composite’s design. The synergistic effect of electrodeposition and Fe coordination modulated the surface topology and chemical composition of GF, constructing a photothermal evaporation interface with high photothermal conversion capability.

**Figure 3:**
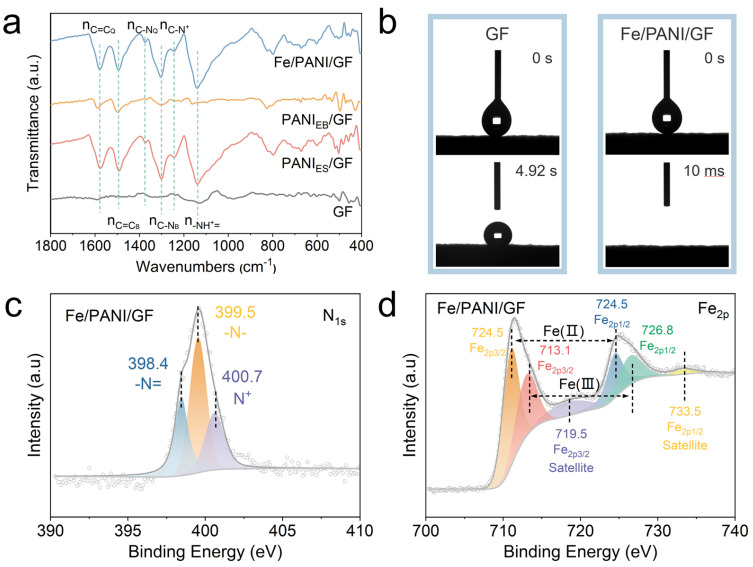
Characterizations of GF composites. (**a**) FT-IR spectra GF, PANIES/GF, PANIEB/GF and Fe/PANI/GF; (**b**) change in contact angle of GF and Fe/PANI/GF; (**c**,**d**) N 1s XPS spectra and Fe 2p XPS spectra of Fe/PANI/GF.

The chemical structure and wettability of the composites were systematically analyzed using FT-IR and contact angle measurements. FT-IR spectra (Figure 3a) revealed no characteristic absorption peaks in the fingerprint region for pristine GF. In contrast, PANI/GF exhibited distinct characteristic peaks at 1560 cm^−1^ (quinoid ring C=C stretching vibration), 1480 cm^−1^ (benzenoid ring C=C stretching vibration), and 1140 cm^−1^ (-NH^+^ = vibration in PANI_ES_/GF), confirming the successful loading of PANI onto the GF surface [24,25,26]. The peak intensity at 1140 cm^−1^ significantly decreased for PANI_EB_/GF after alkali treatment, attributed to the reduction in protonated amine groups due to the dedoping process [20]. For Fe/PANI/GF, the peak intensity at 1140 cm^−1^ was further enhanced compared to PANI/GF, indicating that Fe-N coordination modulated the conjugated structure of PANI through electron transfer, validating the successful incorporation of Fe.

Dynamic contact angle test results (Figure 3b) showed that pristine GF had a contact angle of 125°, exhibiting strong hydrophobicity. Remarkably, Fe/PANI/GF achieved complete water absorption within 10 ms, demonstrating significantly enhanced hydrophilicity. This improvement in wettability is attributed to the exposure of polar groups (e.g., -NH-) in the PANI molecular chains, facilitating rapid water spreading and transport on the material surface, providing a kinetic basis for efficient interfacial evaporation.

High-resolution XPS spectra (Figure 3c,d) further elucidated the chemical states in Fe/PANI/GF. The N 1s spectrum (Figure 3c) was deconvoluted into three peaks: 398.4 eV (-N=, quinoid), 399.5 eV (-N-, benzenoid), and 400.7 eV (N^+^, protonated), consistent with PANI’s emeraldine structure [20,27,28]. The Fe 2p spectrum (Figure 3d) exhibited peaks at 713.1 eV (Fe 2p_3/2_, Fe^3+^) and 726.8 eV (Fe 2p_1/2_, Fe^3+^), along with satellite peaks at 719.5 eV and 733.5 eV, confirming the presence of Fe^3+^ in the composite [29,30]. Additionally, the peaks at 724.5 eV (Fe 2p_3/2_) and 726.8 eV (Fe 2p_1/2_) indicated the coexistence of Fe^2+^, suggesting a mixed-valence state of Fe that may contribute to enhanced photothermal conversion [31,32,33].

Collectively, these characterizations demonstrate that Fe/PANI/GF possesses a well-defined structure, favorable wettability, and tunable Fe chemical states, all of which are critical for its performance in solar-driven interfacial evaporation.

### 3.3. Photothermal Conversion Performance of GF, PANI/GF, and Fe/PANI/GF Composites

**Figure 4:**
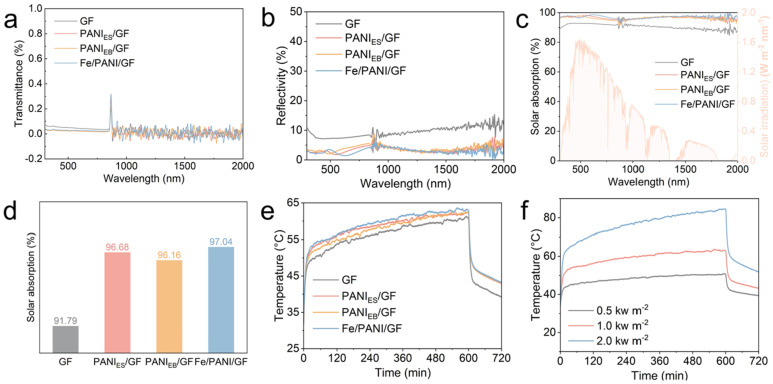
The optical and thermal properties of GF composites. (**a**) Transmittance and (**b**) Reflectivity of various samples; (**c**) *Vis*-NIR spectra and (**d**) solar absorption of various samples in wavelength range of 300–2000 nm range, weighted by standard air mass 1.5 global (AM 1.5G) solar spectrum (red area); (**e**) Photothermal conversion performance of different samples under the solar irradiation of 1.0 kW m^−2^; (**f**) Photothermal conversion performance of Fe/PANI/GF under different light intensities.

The Fe/PANI/GF composite exhibited optimized optical characteristics in the visible to near-infrared (*Vis*-NIR) range (300–2000 nm) (Figure 4a–c). Compared to pristine GF and PANI/GF, its transmittance and reflectance were significantly reduced(Figure 4a,b), indicating that Fe coordination effectively suppressed photon reflection losses by altering the electronic structure of PANI and modulating surface roughness. UV-*Vis*-NIR spectroscopy analysis (Figure 4c,d) showed that the weighted absorption (based on AM 1.5G solar spectrum) of Fe/PANI/GF across the broad spectrum (300–2000 nm) was 5.25% higher than that of GF. Particularly, light absorption in the NIR region (800–2000 nm) was significantly enhanced, confirming its efficient capture of full-spectrum solar radiation.

Photothermal response tests (Figure 4e) demonstrated that under 1.0 kW m^−2^ illumination, the surface temperature of Fe/PANI/GF rapidly increased from 25.3 °C to 60.3 °C within 5 min, reaching a stable value of 63.3 °C at 10 min. This was significantly higher than the temperatures achieved by PANI/GF (61.8 °C) and pristine GF (60.4 °C). When varying irradiation intensities (Figure 4f), Fe/PANI/GF’s temperature increased monotonically with intensity (from 47.4 °C at 0.5 kW m^−2^ to 82.7 °C at 2.0 kW m^−2^), exhibiting excellent intensity-dependent photothermal responsiveness. This performance enhancement is attributed to the synergistic effect between Fe-N coordination and the PANI conjugated system, which enhances light absorption efficiency and achieves efficient solar-to-thermal energy conversion.

### 3.4. Photothermal Evaporation Performance of GF, PANI/GF, and Fe/PANI/GF Composites

**Figure 5:**
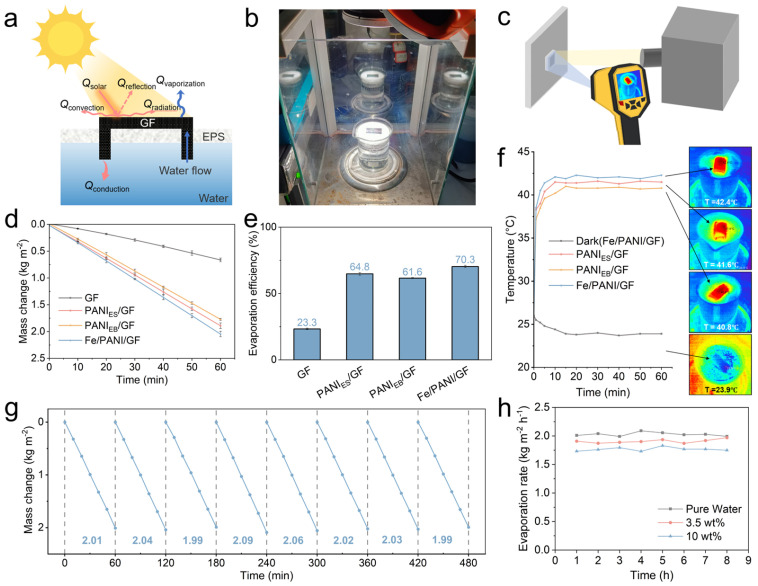
Solar evaporation performance tested at ambient temperature of 25 °C and humidity of 45%: (**a**) Schematic of energy balance during interfacial evaporation, (**b**) Photograph of the experimental setup, (**c**) Schematic of thermal imaging measurement, (**d**) Evaporation rates of different samples under 1.0 kW m^−2^ solar irradiation, (**e**) Evaporation efficiencies of different samples under 1.0 kW m^−2^ solar irradiation, (**f**) Surface temperature profiles and corresponding infrared thermal images of different materials, (**g**) Cyclic mass change in Fe/PANI/GF over multiple evaporation cycles, (**h**) Evaporation rate of water with different salt concentrations.

The solar-driven interfacial evaporation performance of GF, PANI_ES_/GF, PANI_EB_/GF, and Fe/PANI/GF. was comprehensively evaluated, as illustrated in Figure 5.

Figure 5a depicts the energy balance during interfacial evaporation, where incident solar energy (Q_solar_) is distributed among vaporization (Q_vaporization_), conduction (Q_conduction_), convection (Q_conduction_), radiation (Q_radiation_), and reflection (Q_reflection_). The experimental setup (Figure 5b) and thermal imaging measurement configuration (Figure 5c) were employed to quantify water mass loss, evaporation efficiency, and surface temperature, respectively.

The interfacial water evaporation performance of different materials was tested under 1.0 kW m^−2^ solar irradiation. Mass changes were recorded to calculate evaporation rates (Figure 5d). The evaporation rate of pristine GF was only 0.66 ± 0.04 kg m^−2^ h^−1^. PANI_ES_/GF and PANI_EB_/GF exhibited significantly improved evaporation rates of 1.89 ± 0.05 kg m^−2^ h^−1^ and 1.77 ± 0.02 kg m^−2^ h^−1^, respectively, representing increases of approximately 2.8-fold and 2.7-fold compared to GF. The Fe/PANI/GF composite demonstrated the optimal performance, achieving a high evaporation rate of 2.05 ± 0.05 kg m^−2^ h^−1^, which is an 8.6% improvement over the non-coordinated systems (PANI_ES_/GF). Corresponding evaporation efficiencies (*η*) were 23.3 ± 0.54% for GF, 64.8 ± 1.02% for PANI_ES_/GF, 61.6 ± 0.36% for PANI_EB_/GF, and 70.3 ± 0.96% for Fe/PANI/GF (Figure 5e). The 3–8% improvement for Fe/PANI/GF over the single-component PANI/GF indicates that Fe coordination effectively reduced heat conduction losses to the substrate and optimized photothermal conversion efficiency.

Infrared thermal imaging analysis (Figure 5f) revealed that after 5 min of illumination, the surface temperature of Fe/PANI/GF rapidly increased to 42.4 °C and remained stable around 42 °C throughout the test. In contrast, the maximum surface temperatures of PANIES/GF and PANIEB/GF were 41.6 °C and 40.8 °C, respectively. Under dark conditions, the temperature of Fe/PANI/GF slowly decreased to 23.9 °C, a phenomenon potentially associated with the interfacial-assisted evaporation effect of water films adsorbed on the material surface [34,35]. The thermal images further revealed that Fe/PANI/GF formed a more uniform and concentrated high-temperature zone on its surface, indicating superior thermal insulation capability and photothermal conversion homogeneity, providing a guarantee for sustained and efficient interfacial evaporation. Figure 5g demonstrates that Fe/PANI/GF maintained a stable mass loss rate (≈2.0 kg m^−2^ per cycle) over 8 consecutive evaporation cycles, indicating robust durability.

For practical desalination applications, cyclic stability and anti-salt performance are crucial. Additionally, Figure 5h shows that the evaporation rate of Fe/PANI/GF was minimally affected by salt concentrations (3.5 wt% and 10 wt%), with an average decrease of 0.12 kg m^−2^ h^−1^ at 3.5 wt% and 0.26 kg m^−2^ h^−1^ at 10 wt%. This is attributed to the composite’s outstanding wettability and resistance to salt deposition during evaporation.

Collectively, these results confirm that Fe/PANI/GF integrates high evaporation efficiency, rapid temperature rise, cyclic stability, and anti-salt performance, making it a promising candidate for sustainable solar-driven desalination.

## 4. Discussion

This study successfully constructed a novel Fe/PANI/GF composite material via Fe-N coordination and systematically elucidated the structure-performance relationship concerning its solar interfacial evaporation characteristics. The research demonstrates that the PANI/GF composite prepared by electrodeposition significantly enhances the surface roughness and hydrophilicity of graphite felt through nanoparticle coating. Furthermore, Fe coordination synergistically enhances light absorption and modulates the PANI conjugated system, enabling efficient capture of full-spectrum solar radiation (with a 5.25% increase in average NIR absorption) and optimization of photothermal conversion efficiency (achieving a maximum surface temperature 1.6 °C higher than PANI_EB_/GF). Under 1.0 kW m^−2^ solar irradiation, the Fe/PANI/GF composite achieved a high evaporation rate of 2.05 kg m^−2^ h^−1^. This study develops an efficient and economical photothermal material system for solar desalination technology, whose excellent photothermal performance and structural stability demonstrate promising potential for practical applications. Future work should investigate the long-term stability of Fe/PANI/GF under complex water quality conditions and optimize its scalable fabrication process, thereby facilitating the transition from laboratory research to industrial application.

## Data Availability

The original contributions presented in this study are included in the article. Further inquiries can be directed to the corresponding author.

## Abbreviations

The following abbreviations are used in this manuscript:

SDIE	Solar-driven Interfacial Evaporation
PANI	Polyaniline
GF	Graphite felt
